# Case report of selumetinib as a novel therapy in a neurofibromatosis type 2-associated ependymoma

**DOI:** 10.1016/j.omtm.2023.101156

**Published:** 2023-11-10

**Authors:** Nigel Blackwood, Christopher Zetzmann, Christopher R. Trevino

**Affiliations:** 1 Tulane University School of Medicine, 1430 Tulane Avenue, New Orleans, LA 70112, USA; 2 Tulane University School of Medicine, Department of Radiology, 1415 Tulane Avenue, New Orleans, LA 70112, USA; 3 Tulane University School of Medicine, Department of Medicine/Section of Hematology-Medical Oncology, 1430 Tulane Avenue, #8078, New Orleans, LA 70112, USA

**Keywords:** neurofibromatosis 2, selumetinib, MEK inhibitor, ependymoma, SMARCB1

## Abstract

We report partial response (PR) to novel therapy with selumetinib in a patient with neurofibromatosis type 2 (NF2). A 25-year-old male presented with bilateral vestibular schwannomas, spinal cord intramedullary ependymomas, cranial and spinal meningiomas, spinal nerve root mixed schwannoma-neurofibromas, and peripheral nerve sheath tumors. He tested negative for germline *NF2*, SWItch/sucrose non-fermentable-related matrix-associated actin-dependent regulator of chromatin subfamily B member 1 (*SMARCB1)*, and leucine zipper-like transcription regulator 1 (*LZTR1)* mutations. Molecular analysis of a resected cervical spine schwannoma-neurofibroma demonstrated an isolated somatic *SMARCB1* mutation. Due to progression of all tumors, he was treated medically with both everolimus (10 mg/day) and selumetinib (25 mg/kg twice a day), but he rapidly transitioned to selumetinib monotherapy due to everolimus toxicity. 3 months of treatment resulted in PR in one spinal ependymoma and stable disease in other tumors. This PR was quantified by the differences in units of intensity in pre- and post-treatment magnetic resonance image. To the best of our knowledge, this is the first reported case for using selumetinib in NF2-associated tumors or ependymomas.

## Graphical abstract

**Figure:**
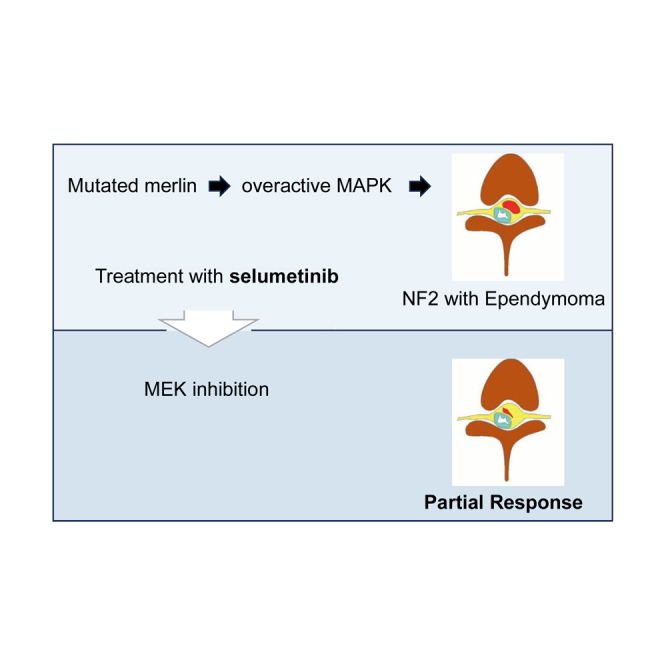

## Teaser

A 25-year-old male with NF2 treated with selumetinib had a partial response in one spinal ependymoma and stable disease in other tumors at 3 months. This partial response was quantified by pre- and post-treatment magnetic resonance image units of intensity. This is the first reported case for using selumetinib in NF2 or ependymomas.

## Introduction

Neurofibromatosis type 2 (NF2) is diagnosed by either clinical or genetic criteria. Clinical diagnostic criteria include bilateral vestibular schwannomas, meningiomas, ependymomas, spinal schwannomas and juvenile cataracts. By genetic criteria, NF2 occurs due to a mutation in the *NF2* tumor suppressor gene encoding the merlin protein. Defective merlin protein leads to overactivation of the Ras/Raf/mitogen-activated protein kinase kinase (MEK)/mitogen-activated protein kinase (MAPK) pathway, in addition to overactivating a parallel phosphatidylinositol 3-kinase (PI3K)/protein kinase B(AKT)/mammalian target of rapamycin (mTOR)/signal transduction and activator of transcription (STAT3) pathway.

Although recent recommendations for genetic diagnosis of NF2 included exclusion of SWItch/sucrose non-fermentable-related matrix-associated actin-dependent regulator of chromatin subfamily B member 1 (*SMARCB1)*, SWItch/sucrose non-fermentable-related matrix-associated actin-dependent regulator of chromatin subfamily E member 1 (*SMARCE1)*, and leucine zipper-like transcription regulator 1 (*LZTR1)* with a goal of avoiding misdiagnosis of schwannomatosis or meningiomatosis, the scientific community is changing after identifying increasing molecular heterogeneity among NF2 patients with expanded gene evaluation. A comprehensive French study genetically profiled NF2 patient blood and tumor DNA and revealed additional mutations in *SMARCB1* and *LZTR1* in 65% of patients.^1^^,^^2^ *SMARCB1* is a tumor suppressor gene involved in chromatin uncoiling and remodeling. Mutations in *SMARCB1* are associated with atypical teratoid/rhabdoid tumors (AT/RT) in infants and schwannomatosis in adults. Studies suggest *LZTR1* suppresses tumorigenesis by breaking down unneeded proteins.

Therapy for NF2 generally includes monitoring or symptomatic tumor treatments, e.g., surgical excision or radiation for tumor bulk causing mass effect. Hearing aids, auditory implants, and physical therapy assist symptom management.

The blood-brain barrier (BBB) frequently limits central nervous system (CNS) penetration of drugs, which presents a challenge in effectively treating tumors of the brain and spine. For hydrophilic molecules, this is primarily mediated by the drug efflux transporters P-gp and breast cancer resistance protein, among others. Murine models to evaluate the pharmacokinetics of selumetinib demonstrate limited CNS penetration with very low brain-to-plasma ratios of 0.02.^3^^,^^4^ Murine models also demonstrate everolimus to have poor CNS penetration with dose-dependent low brain-to-plasma ratio ranging from 5.3 to 19.9. P-gp efflux transporters are hypothesized as the culprit. Everolimus carries a long half-life in CNS tissue as well as locally increased penetration due to BBB breakdown at sites of tumor neovascularization.^5^

We present a case of medical therapy for NF2-associated tumors with everolimus and selumetinib. Everolimus is an mTOR pathway inhibitor that has been FDA approved for vestibular schwannomas in patients with NF2.^6^^,^^7^^,^^8^^,^^9^ Selumetinib is an MEK pathway inhibitor that has been FDA approved for plexiform neurofibromas in patients with neurofibromatosis type I.^10^^,^^11^ To the best of our knowledge, there is no published literature for the use of selumetinib for treating tumors in patients with NF2.

## Case report

**Figure 1:**
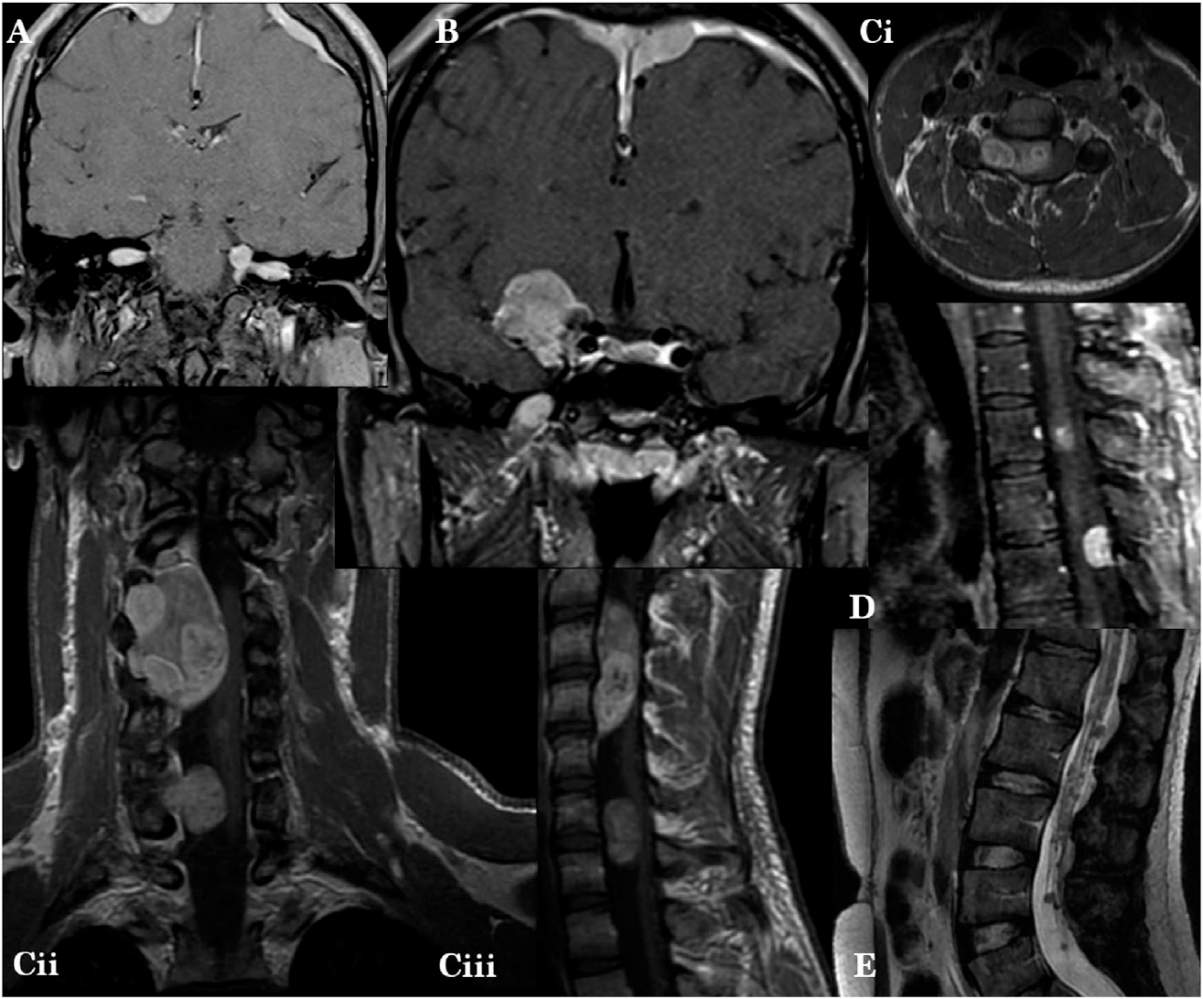
Multiple inherited schwannomas, meningiomas, and spinal ependymomas (A) Coronal T1 W + C image demonstrates enhancing masses obliterating the internal auditory canal cerebrospinal fluid (CSF) spaces, on the left in the hallmark configuration of a so-called “ice-cream scoop” (CPA) on a “cone” (IAC), compatible with vestibular schwannomas. (B) Avidly enhancing dural-based masses along right sphenoid ridge and falx cerebri, compatible with meningiomas. The parafalcine right frontal lesion also features a classic dural “tail” with CSF cleft. (Ci–iii) Biopsy-proven schwannoma-neurofibromas with mass effect compressing the cervical spinal cord. (D) Thoracic cord tumors most consistent with ependymomas that demonstrate patchy post-contrast enhancement. Index lesion is shown at the level of T3, superiorly, with an enhancing posterior meningioma at T5. (E) Innumerable tiny lesions speckle the cauda equina, consistent with spinal nerve sheath tumors.

A 17-year-old male with no significant past medical history initially developed binocular diplopia and impaired eyelid closure. Over the course of an 8-year workup to age 25, he was found to have multiple tumors throughout the neuro-axis, as depicted in Figure 1. MRI (magnetic resonance image) showed bilateral vestibular schwannomas (Figure 1A). Enhancing extra-axial tumors with characteristic imaging appearance of meningiomas were found along the right sphenoid wing and parafalcine right frontal region (Figure 1B). Mixed biopsy-proven schwannoma-neurofibroma was found in the right C3–C7 nerve roots, with mass effect expanding into the spinal canal and compressing the cord at C3–C4 (Figure 1Ci–iii). Intramedullary tumors most likely representing ependymomas were seen at C1, C5–C6, T3, T6, T7, and T8 (Figure 1Ci–iii). Classic meningioma was found posterior to T5 (Figure 1D). Innumerable peripheral nerve sheath tumors most consistent with multiple schwannomas were scattered along the cauda equina (Figure 1E). A lumbosacral plexus tumor concerning for mixed schwannoma-meningiomas is not depicted in the figure.

**Figure 2:**
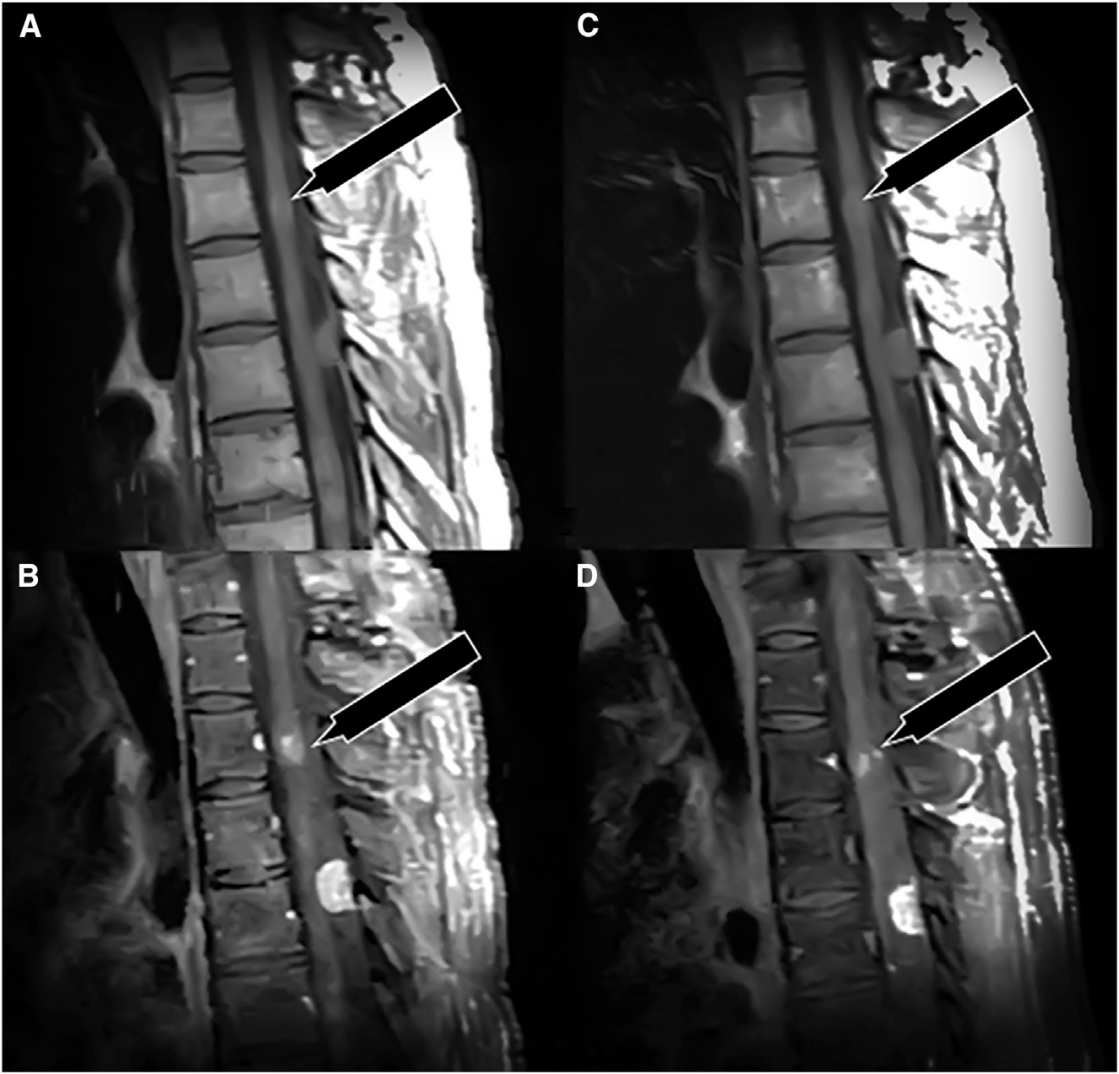
Response to 3-month selumetinib treatment Pre- (A, B) and post-treatment (C, D) thoracic spine MRIs at 3 months. (A) Pre-treatment non-contrast sagittal T1-weighted images of the thoracic spine demonstrate multifocal spinal cord signal alteration and subtle contour abnormality at the level of T3. An extramedullary posterior spinal canal lesion at the level of T5 is also seen. (B) Same-date sagittal T1 fat-saturation post-contrast images reveal avid enhancement in these tumors. The intramedullary tumors, including the T3 level lesion, are consistent with spinal ependymomas. The extramedullary T5 lesion with dural tail has imaging characteristics of meningioma. (C) Post-treatment non-contrast images taken at 3 months of treatment demonstrate similar distribution and characteristics of these same tumors. (D) Note the visible decrease in conspicuity of the T3 ependymoma, correlating with a measurable decrease in enhancement compared to pre-treatment images, compatible with a partial response. The T5 lesion persistently enhances. Special note: contrasted MRIs were taken after intravenous administration of gadolinium-based contrast material. Images were acquired at the same institution on the same scanner utilizing the same protocol. Regions of interest were drawn to include the tumors at the same level in the midline covering 50% of the total area. Standardized comparison of pre- and post-treatment images was performed on licensed post-processing software. “Enhancement” was standardized as a change in intensity after contrast administration of greater than or equal to Δ15. The T3 ependymoma lesion prior to treatment minimally enhanced (pre- and post-contrast intensity difference = Δ20). On 3-month post-treatment images, the lesion does not meet the threshold for enhancement (Δ3). For comparison and internal control, the T5 meningioma demonstrates significant pre and post-treatment post-contrast enhancement (Δ232 and Δ202, respectively). Thus, we cautiously support our claim of partial response with reproducible data.

1 year after initial diagnosis, he was found to have cervical spinal cord compression requiring tumor resection as well as a C3–C7 posterior laminectomy with left C3–T1 instrumented fusion. The pathology demonstrated a mixed schwannoma-neurofibroma, and sequencing identified a *SMARCB1* mutation with wild-type *NF2* gene. 7 years after initial symptoms, he had a left frontal craniotomy for a subtotal meningioma resection. He met clinical criteria for NF2 from his tumor types and underwent germline genetic testing using a saliva sample for *NF1*, *NF2*, *LZTR1*, and *SMARCB1*, which did not demonstrate any mutations. Given the extensive tumor burden, multiple tumor types, and inability to resect all the lesions in his spinal cord, treating physicians recommended medical treatment rather than surgery. He initially began treatment with everolimus (10 mg/day) and selumetinib (25 mg/kg twice a day [BID]) with a goal of blocking the parallel driving pathways in NF2. He discontinued both treatments after 2 weeks due to Common Terminology Criteria for Adverse Events (CTCAE v5.0) grade III oral mucositis and grade III maculo-papular rash. 2 weeks later, the patient resumed selumetinib (25 mg/kg BID) monotherapy with variable adherence for 3 months. His repeat neuroaxis imaging demonstrated stable disease in nearly all tumors, but he demonstrated a partial response (PR) in the T2 intramedullary tumor concerning for a spinal ependymoma (Figure 2). Diagnostic images were standardized by a radiologist and showed qualitatively reduced contrast enhancement in post-treatment images, which is compatible with a PR. This PR was quantified by the differences in units of intensity in pre- and post-treatment MRIs (Figure 2). Unfortunately, the patient chose to discontinue systemic therapy due to mild fatigue and CTCAE grade II skin rash despite topical steroids and oral doxycycline.

## Discussion

This patient demonstrates clinical hallmarks of NF2 such as bilateral vestibular schwannomas, ependymomas, and meningiomas. However, germline testing was negative for *NF2* mutation, and sequencing of tumors revealed wild-type *NF2*. Somatic next-generation sequencing of the spinal schwannoma-neurofibroma showed an isolated *SMARCB1* mutation, suggesting a possible unique somatic oncologic driver. Although uncertain, the negative germline testing for *SMARCB1* may have been a false negative due to sampling error from saliva testing.

Most somatic mutations for tumors in patients with NF2 harbor homologous loss of *NF2* and sometimes carry a concomitant *SMARCB1* or *LZTR1* mutation.^12^ However, this was not present in our case.

The mechanisms underlying familial cancer syndrome remain unclear as a multitude of gene mutations are associated with these diseases. *SMARCB1* mutation-driven AT/RTs demonstrate response to MEK inhibitors, suggesting *SMARCB1* may cause overactivation of MAPK pathway.^13^

After medical treatment with selumetinib, MRIs demonstrated multiple stable tumors and a PR in one intraspinal tumor that we suspect is an ependymoma by imaging. Despite the limited CNS penetration of selumetinib, it is possible the local BBB breakdown in the tumor’s microvasculature could increase local penetration. Currently, there is an ongoing pediatric clinical trial using selumetinib for tumors associated with children with NF2 based on pre-clinical data (NCT03095248). The tumors under investigation include vestibular schwannomas, meningiomas, ependymomas, and gliomas.

To the best of our knowledge, this is the first reported case with a PR in NF2-associated tumors or in an ependymoma. We present this case as an example of the increasing role of targeted therapies in familial cancer syndromes. The role of MEK inhibitors and other targeted therapies in CNS tumors warrants further investigation in clinical trials.
